# Differential Performance of Children and Adults in a Vision-Deprived Maze Spatial Navigation Task and Exploration of the Impact of tDCS over the Right Posterior Parietal Cortex on Performance in Adults

**DOI:** 10.3390/life15081323

**Published:** 2025-08-20

**Authors:** G. Nathzidy Rivera-Urbina, Noah M. Kemp, Michael A. Nitsche, Andrés Molero-Chamizo

**Affiliations:** 1 Faculty of Administrative and Social Sciences, Autonomous University of Baja California, Blvd. Juan A Zertuche y Blvd de los Lagos s/n Fracc. Valle Dorado, Ensenada C.P. 22890, Baja California, Mexico; noah.kemp@uabc.edu.mx; 2 Leibniz Research Centre for Working Environment and Human Factors, 44139 Dortmund, Germany; 3 University Clinic of Psychiatry and Psychotherapy, Protestant Hospital of Bethel Foundation, University Hospital OWL, Bielefeld University, 33602 Bielefeld, Germany; 4 German Centre for Mental Health (DZPG), 44787 Bochum, Germany; 5 Department of Psychology, University of Huelva, 21071 Huelva, Spain; andres.molero@dpsi.uhu.es

**Keywords:** adults, children, maze task, posterior parietal cortex, spatial navigation, tDCS

## Abstract

Spatial navigation involves the use of external (allocentric) and internal (egocentric) processing. These processes interact differentially depending on age. In order to explore the effectiveness of these interactions in different age groups (study 1), we compared the performance of children and adults in a two-session spatial maze task. This task was performed under deprived vision, thus preventing visual cues critical for allocentric processing. Number of correct performances and performance time were recorded as outcome measures. We recruited thirty healthy participants for the children (mean age 10.97 ± 0.55) and the adult (mean age 21.16 ± 1.76) groups, respectively. The results revealed a significantly higher number of correct actions and shorter performance times during maze solving in children compared to adults. These differences between children and adults might be due to developmental and cortical reorganization factors influencing egocentric processing. Assuming that activation of the posterior parietal cortex (PPC) facilitates egocentric spatial processing, we applied excitatory anodal tDCS over the right PPC in a second study with a different healthy adult group (*N* = 30, mean age 21.23 ± 2.01). Using the same spatial navigation task as in study 1, we evaluated possible performance improvements in adults associated with this neuromodulation method. Compared to a sham stimulation group, anodal tDCS over the right PPC did not significantly improve spatial task performance.

## 1. Introduction

Spatial orientation and navigation processes require the identification of multimodal sensory stimuli and complex cognitive processing [1,2,3,4,5]. Spatial processing relies on a combination of two orientation strategies described as allocentric and egocentric processing. Allocentric spatial processing, also known as place learning or cognitive mapping, is an external reference strategy involving learning, memorizing, and recalling the location of objects in a specific space, supported by an internal representation of the environment (cognitive map) [6,7]. This processing is therefore critically dependent on visual references. By contrast, in egocentric spatial processing, the organism itself becomes a reference point within the surrounding space. The egocentric strategy relies on the integration of visual, kinesthetic, and vestibular stimuli to perceive movements, turns, distances, and directions in external space [2,3,6]. Functional neuroimaging studies point to an activation of the posterior parietal-frontal network during egocentric processing, while the posteromedial-temporomedial cortical network seems to be involved in allocentric processing [2,3,8,9].

The neural networks involved in egocentric processing begin to develop in childhood [10,11], during critical developmental periods that facilitate cortical reorganization [12], and functional maturation is associated with the structural maturation of the nodes that make up these networks. In the case of the parietal lobe, maturation processes are prominent at the age of 11–13 years [13,14]. In contrast, the external reference strategy based on allocentric processing develops in later stages of maturation, becoming the prevalent strategy in adulthood [15]. Environment-centered allocentric navigation is therefore considered a high-level cognitive ability. In contrast, allocentric processing is restricted in children due to the limited ability to use spatial landmarks for navigation [16].

Once allocentric processing has matured in adulthood, orientation and navigation processes in space require continually updating sensory and motor information [17]. The main external sensory input during spatial navigation are visual stimuli, probably in interaction with other cues and sensory stimuli specific to the spatial context. However, blind people perform spatial orientation tasks effectively in the absence of visual input via egocentric navigation [12,18,19], and even certain spatial tasks can be correctly performed by sighted people who are deprived of vision during task performance. When vision is impaired or deprived, the age-dependent prevalence of allocentric/egocentric processing can be modulated in adults. The contribution of proprioceptive, kinesthetic, and other sensory modalities can be enhanced in adults under visual deprivation, so that the cognitive processes of navigation might rely on these other sensory inputs during spatial processing [20,21]. The effective triggering of these compensatory processes and egocentric strategies in adults may ultimately depend on the ability to adapt to contexts of sensory restriction. Therefore, it would be expected that performance in spatial tasks under vision deprivation would differ between age groups, assuming that visual dependence (allocentric) and compensatory cognitive processes (egocentric) are not equally involved in children and adults.

The place cells of the dorsal hippocampus [22], which respond to the orientation of the external environment and specific spatial locations (allocentric processing), provide the basis for cognitive maps [23,24]. The posterior parietal cortex (PPC) is another crucial area for spatial navigation. This node of the spatial processing network serves as a multimodal association cortex, integrating inputs from somatosensory, auditory, visual, motor, cingulate, and prefrontal cortices. Additionally, the PPC receives projections from subcortical areas involved in proprioceptive and vestibular processing [25]. The PPC integrates these inputs to create an internal or egocentric framework of reference that collates the information needed, via the posterior parietal-frontal network, to coordinate goal-directed response systems [24,26].

In adults, neuromodulation of the PPC might facilitate spatial processing under conditions of visual deprivation, either by reducing the visual dependence of cognitive processes or by enhancing the efficacy of other sensory inputs and, eventually, of compensatory cognitive processes. Through any of these mechanisms, activation of the PPC might promote a shift to egocentric spatial processing strategies that would facilitate spatial navigation without visual input. Transcranial direct current stimulation (tDCS) is a non-invasive neuromodulation method with proven capacity to modulate cortical excitability [27] and potentially underlying functions [28]. In the context of spatial egocentric processing, it has been shown that excitatory anodal tDCS over the right PPC enhances cognitive processes underlying spatial navigation [29]. Additionally, fMRI studies have revealed that anodal tDCS over the medial parietal cortex (Pz EEG position) may enhance functional connectivity during spatial navigation and allocentric processing [30,31]. Application of anodal tDCS over the left PPC (P3 EEG position) has been associated with memory consolidation in implicit visuo-motor learning tasks [32,33,34], revealing the multimodal involvement of the parietal cortex in visuospatial recognition processes and egocentric processing. Moreover, anodal tDCS targeting the right temporoparietal junction seems to be a promising therapeutic approach for restoring spatial navigation processes and improving spatial memory deficits in patients with Alzheimer’s disease [35].

Hypothesizing that there are differences in spatial navigation under visual deprivation between children and adults and that activation of the PPC may facilitate egocentric processing in adults under limited access to allocentric inputs [36], we formulated two research objectives (study 1 and 2). These objectives were aimed at (i) analyzing performance in different age groups under restriction of allocentric processing and (ii) evaluating the possibility to improve performance under neuromodulation. First, we aimed to explore possible age-related differences in performance of a maze spatial navigation task under vision deprivation (study 1). For this purpose, we compared two different age groups in this study: children and young adults. Assuming a different influence of allocentric (environment-centered and focused on visual stimuli) and egocentric (self-centered and based on internal cognitive references) processing during spatial navigation between children and adults [15], we expect less dependence on allocentric visual input during spatial navigation task performance in children, which would result in better performance in this age group under visual deprivation. In study 2, using the same navigation task, we aimed to increase the activity of the PPC in order to facilitate egocentric processing in adults under limited access to allocentric inputs (deprived vision). As a critical node of the spatial processing network sensitive to internal egocentric cues, we focused on this cortical target versus other possible nodes with potentially less control over this function, such as sensorimotor cortical areas or medial temporal regions. We expect improved performance in adults under excitatory neuromodulation of the PPC, in line with previous studies [29].

## 2. Study 1

### 2.1. Materials and Methods

#### 2.1.1. Participants

Sample size calculation was based on the hypothesized significant differences for the between-participant-factor age (children vs. adults), and the within-group-factor session for the dependent variable task performance. We conducted an a priori calculation of the sample size by the GPower statistical power analysis tool (3.1.9.2) for a mixed model repeated measures ANOVA (with groups and session measures as levels of the between-group and within-group factors) [37]. We conducted the analysis for the within–between interaction, setting the following input parameters: effect size (f) = 0.25 (medium effect size adjusted for preliminary results), α value = 0.05, Power (1 − ß) = 0.95, number of groups = 2 (children vs. adults), number of measurements = 2 (sessions 1 and 2), correlation for repeated measures = 0.5, and nonsphericity correction (1). The output parameters were the following: non-centrality parameter = 13.5, critical F = 4.02, actual power 0.95, and total sample size = 54. Taking into account these estimates and adding 15% to the calculated sample to compensate for possible dropouts, the total estimated sample size is 62.

According to these calculations, the total sample size finally included 30 healthy children (15 girls and 15 boys, mean age 10.97 ± 0.55 years) and 30 healthy adults (15 women and 15 men, mean age 21.16 ± 1.76 years), recruited through advertisements posted on the Autonomous University of Baja California website. There were no withdrawals from the study, and analyses were performed for all sample participants. According to previous interviews, the socioeconomic status and educational background were comparable between participants in each sample (Table 1). No participant had prior experience with spatial navigation tasks. All adults provided informed written consent before inclusion in the study, and the children’s parents provided written authorization for their children’s voluntary participation, who previously agreed explicitly to their participation in this study. The study was approved by the Ethics Committee of the Autonomous University of Baja California and conformed to the principles of the World Medical Association Declaration of Helsinki.

#### 2.1.2. Maze Spatial Navigation Task

**Figure 1:**
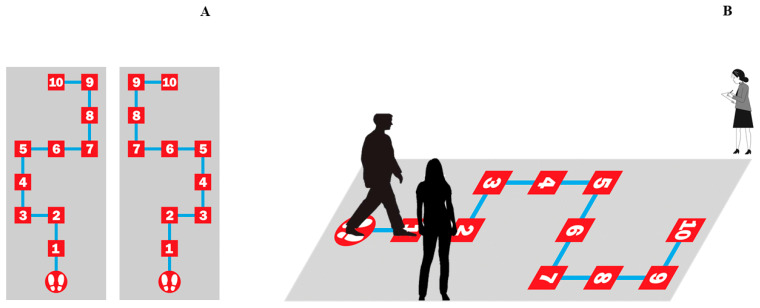
Spatial navigation task in a physical maze. (**A**). Outcome measures were obtained in two sessions. All participants performed two versions of the task one week apart between each version, in counterbalanced order across participants. In order to balance the difficulty of both versions of the task, the routes were mirror images of each other. (**B**) In each version, a different 10-step route was shown and performed by one of the researchers before the start of the task. Then, participants were visually deprived by opaque glasses. During task performance, the research staff provided verbal feedback about success or failure at each step (right, left, or straight ahead) and helped participants to return to the starting point after each wrong step. The task ended after 20 unsuccessful trials. The number of correct performances in each session and performance time were recorded by a second member of the research staff.

In order to record a sufficient number of responses at different measurement times, all participants performed two sessions of a maze spatial navigation task (MSNT) one week apart. A different version of the task was used in each session in counterbalanced order across participants. The MSNT consisted of a ten-step route, each consisting of a turn to the right, left, or straight ahead. The two versions were mirror images of each other (Figure 1), thus providing two versions that were only different in 3 of the 10 steps of the maze (turn left or right, depending on the version), resulting in objectively different, but highly comparable versions with respect to difficulty level. At the beginning of each session, participants visualized the route for 60 s, before being vision-deprived to perform the task. Visual deprivation was performed using opaque protective glasses (fully sealed). During task performance, participants received verbal feedback on whether each step (right, left, or straight ahead) was correct or not. In case of a mistake, participants returned to the starting point to start the task again. After 10 attempts, if a participant was unable to complete the task, he/she was allowed to view the route again for another 60 s. After 15 trials without successfully completing the task, participants were allowed to view the route again for another 60 s. If the task was not correctly completed after 20 trials, the session was ended and considered incomplete (no correct performance) (Figure 1). Given the lack of standardized physical spatial tests and references, we considered this a reasonable number of trials to determine successful performance on the task without exhausting and compromising the attentional and cognitive systems necessary for task performance. Total performance time in each session and correct performance (defined as correctly reaching position 10 of the maze route) were recorded in each case for analyses.

#### 2.1.3. Data Analysis

Total performance time until successful (reaching position 10 of the route) or incomplete (not reaching position 10 after 20 trials), performance of the task, and the number of successful performances in each group were analyzed separately as dependent variables. A mixed repeated-measures ANOVA, with group (children vs. adults) as between-subject factor and session (1 and 2) as within-subject factor, was conducted to analyze differences in the dependent variables between groups and between sessions. Independent-samples and paired-samples *t*-tests were performed to compare between-subject and within-subject values, respectively, in case of significant results of the ANOVA. To control for type I errors, Bonferroni corrections were applied to these comparisons. The critical level of significance in all tests was set to *p* < 0.05. Data were analyzed using the IBM SPSS v24 software (IBM CORP, Armonk, NY, USA).

### 2.2. Results

**Figure 2:**
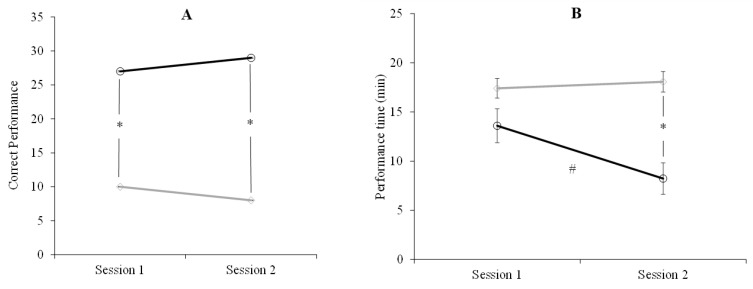
Spatial navigation task performance of children (○) and adults (◊). (**A**) Number of correct performances of the task. (**B**) Performance time in minutes. (*) Significant differences between groups. (#) Significant differences between sessions (session 1 and 2). Error bars represent standard error of means (SEM). These results indicate that children perform the spatial task correctly at a higher rate than adults in both sessions. Furthermore, children took less time to complete the task compared to adults, and, additionally, they improved their performance times in the second session.

A repeated-measures ANOVA revealed that children outperformed adults across sessions (F_1,58_ = 29.8, *p* < 0.001, *ηp*^2^ = 0.340). Post-hoc comparisons with Bonferroni corrections showed that the number of correct performances was significantly higher in children than in adults in sessions 1 (*d* = 1.0 *p* = 0.008) and 2 (*d* = 1.0, *p* < 0.001). There was no significant effect of the factor session (F_1,58_ = 0.01, *p* = 1.0, *ηp*^2^ = 0.01) or the interaction between the factors group and session (F_1,58_ = 1.126, *p* = 0.293, *ηp*^2^ = 0.019) (Figure 2A).

The ANOVA conducted for performance time showed that children took less time than adults (F_1,58_ = 16.637, *p* < 0.001, *ηp*^2^ = 0.223). The factor session was also significant (F_1,58_ = 4.849, *p* = 0.032, *ηp*^2^ = 0.077), with times being shorter in session 2 (*d* = 0.78, *p* < 0.001). The interaction between group and session was significant as well (F_1,58_ = 7.990, *p* = 0.006, *ηp*^2^ = 0.077), with improvements in session 2 only in children (*d* = 1.0, *p* = 0.014) (Figure 2B).

## 3. Study 2

Since study 1 showed that adults performed worse than children, we conducted a second study to assess whether stimulating the PPC can enhance spatial performance in adults.

### 3.1. Material and Methods

#### 3.1.1. Participants

We recruited participants via advertisements posted on the same institution’s website as in study 1. We used the same sample size per group as in study 1. Thus, thirty adults (a single group, with 15 women, mean age 21.23 ± 2.01) who did not participate in study 1, voluntarily participated in study 2. There were no dropouts during the study, and analyses were conducted for all participants. No relevant socioeconomic or educational differences were found between participants (Table 1). No participant had prior experience with spatial navigation tasks. All of them provided written informed consent before inclusion in the study. Participants were not assigned to different groups, as a crossover design was used in which all participants received all stimulation conditions. None of the participants were under treatment with psychoactive medication, reported any previous or current neurological or psychiatric diseases, or met the exclusion criteria typically described in tDCS studies (metal in any part of the head, pacemaker, cranial fissures or holes, etc.) [38]. The research protocol was approved by the Ethics Committee of the Autonomous University of Baja California and conformed to the principles of the World Medical Association Declaration of Helsinki.

#### 3.1.2. Maze Spatial Navigation Task

As in study 1, all participants performed two versions of the MSNT, each in a different session. The order of the task version and stimulation condition was fully counterbalanced across participants.

#### 3.1.3. Transcranial Direct Current Stimulation (tDCS)

tDCS was administered using a battery-driven constant-current stimulator (TCT Research tDCS Stimulator, TST Kowloon, Hong Kong) [39], with conductive rubber electrodes placed between saline-soaked sponges. The anode and cathode electrode size, targeting the right PPC and the left supraorbital ridge, respectively, was 20 cm^2^ (4 × 5 cm) and 35 cm^2^ (7 × 5 cm). As a proxy of the anatomical position of the right PPC, the anode electrode was placed over the P4 position according to the international 10–20 EEG system, based on individual head measurements [40]. The size of the electrode positioned over the PPC (anode) was slightly smaller than that of the return electrode (cathode) so that the current density was larger over the target region [41,42]. The electrodes were fixed onto the head by a tDCS headstrap (CMUS1209, Caputron Universal Strap, New York, NY, USA). Stimulation was applied during task performance at 1.5 mA current intensity (anode: 0.075 mA/cm^2^ and cathode: 0.042 mA/cm^2^ current density at the electrode–skin interface), with gradual increase and decrease for 8 s at the beginning and the end of stimulation, respectively. Current intensity, electrode size, current density, and stimulation duration were within the standard stimulation safety parameters used in conventional tDCS studies [43,44,45]. Similar stimulation protocols resulted in excitability changes stable for about 1 h after motor [27,46] and parietal [47] cortex tDCS in previous studies. For sham tDCS, current was increased and then decreased over 8 s at the beginning and the end of stimulation, but no current was applied for the remainder of the session. After each tDCS intervention, participants were verbally asked to report any sensation experienced during stimulation (tingling, itching, slight warmth on the scalp area under each electrode). Tingling sensations associated to tDCS were reported in both stimulation conditions (recorded as the presence or absence of this effect), but no other side effects were consistently observed. No serious adverse effects during or after the study sessions were reported. Participants were also asked for the stimulation condition (anodal vs. sham) they thought they received.

SimNIBS 4.1.0 (Simulation of Non-Invasive Brain Stimulation), a software based on the finite element method for simulation of electric fields [48] was used to calculate and model the intensity and distribution of the cerebral electric field induced by the electrode configuration described above. The SimNIBS magnetic resonance image (MRI) coordinates (axis x, y, z) for the P4 electrode position were as follows: x = 52.51; y = −55.47; z = 52.37. The coordinates for the cathodal return electrode position, corresponding to the Fp1 EEG position, were as follows: x = −26.59; y = 113.09; z = 19.35. These coordinates correspond to the specific MRIs used by this tool to create a head model by which the cortical regions of interest can be localized via the EEG electrode positioning system. SimNIBS default tissue isotropic conductivity values for scalar brain anisotropy are 0.126 S/m for white matter, 0.275 S/m for gray matter, 1.654 S/m for cerebrospinal fluid (CSF), 0.010 S/m for the bone (skull), 0.465 S/m for the scalp, 0.5 S/m for the vitreous bodies of the eyes, and 1.0 S/m for the saline-soaked sponges [49].

#### 3.1.4. Procedure

All participants completed the same maze spatial navigation task described in study 1. tDCS was applied continuously during task performance. Stimulation was stopped once the task was completed, with an average time of 18.2 ± 5.75 min after 20 unsuccessful trials. The tDCS device was carried during task performance in a small bag held from the shoulder. All participants underwent anodal and sham tDCS and performed two versions of the task in counterbalanced order one week apart. Since no significant performance differences were found between sessions for the adult group in study 1, in study 2 we used the same versions of the task, each one in each stimulation condition (anodal and sham). A double-blind procedure was used to exclude potential intervention biases in each group. Subjects were blinded to the tDCS condition, and a researcher not involved in the data analyses programmed the stimulation condition in each session according to the counterbalancing criterion. Performance time and correct performance in each session were recorded and analyzed as outcome measures as described in study 1.

#### 3.1.5. Data Analysis

As in study 1, total performance time in each session and number of correct performances were analyzed separately as dependent variables. A one-way ANOVA was conducted to analyze differences between stimulation conditions (anodal vs. sham tDCS) for each dependent variable. The chi-square test (χ^2^) was conducted to analyze possible differences in percentages of correct identification of the tDCS condition (anodal vs. sham), as well as the percentage of presence of tingling reported by each group. The critical level of significance in all tests was set to *p* < 0.05. Data were analyzed using the software IBM SPSS v24 software (IBM CORP).

### 3.2. Results

**Figure 3:**
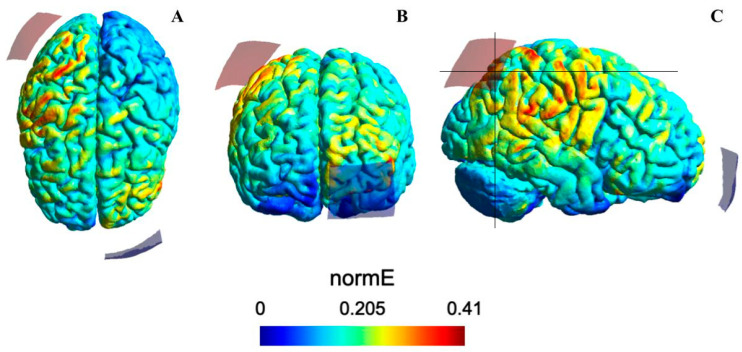
Intensity of the electric field induced by tDCS applied over the right posterior parietal cortex (PPC, corresponding to the P4 EEG electrode position). The images from left to right show, respectively, a dorsal (**A**), frontal (**B**), and lateral (**C**) view of the brain. The electric field (normative strength: normE) intensity (V/m) is represented by the color bar. The red colors (larger number depicted in the color bar) indicate higher electric field intensity—with the highest intensity corresponding to the right parietal cortex and, particularly, the PPC region (intensity values within the red band of the color bar, panel (**C**))—and a considerable loss of intensity from the regions adjacent to the cortical target. SimNIBS 4.1.0 (Simulation of Non-Invasive Brain Stimulation) was used for modeling the electric field. Red and blue electrodes of the SimNIBS output brain images represent the anodal and cathodal electrode positions, respectively. These positions should not be considered as a direct position over the brain targets, considering the anatomical space between brain and electrode positions over the head.

The simulation of the intensity and distribution of the electric field associated to the electrode configuration used in the present study using the SimNIBS software with the P4 area as cortical target showed that the highest electric field intensities (>0.3 and <0.41 V/m) were located in the right parietal cortex, including the PPC (area in red shown in Figure 3C). Although the maximum electric field strength over the cortical target does not guarantee effective stimulation at the neural level, the modeling provides reliable estimations of the stimulation intensities whose values have been associated with changes in cortical excitability [50,51].

No serious adverse effects during or after the application of tDCS were reported in any group. The occurrence of tingling was the most frequently reported adverse effect in both stimulation conditions. The chi-square test [χ^2^ = 30 (1, *N* = 30)] showed that this effect was reported to a similar amount in the anodal and sham stimulation conditions (55% of reports in the anodal condition vs. 49% in sham, *p* > 0.05). No other adverse effects were consistently reported. According to the chi-square test results [χ^2^ = 0.10 (1, *N* = 30)], there were no between-group differences in the proportion of guessing of anodal and sham stimulation. Fifty-four percent of participants in the anodal group and fifty-one percent of participants in the sham group estimated that they received anodal tDCS. Forty-six percent and forty-nine percent of participants in the anodal and sham groups estimated, respectively, that they received sham stimulation. Therefore, the stimulation conditions could not be reliably discerned in both groups.

**Figure 4:**
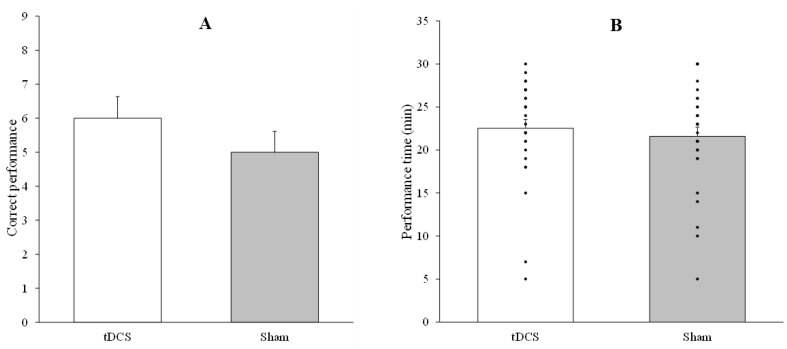
Spatial navigation task performance of adults under right posterior parietal cortex anodal vs. sham tDCS. (**A**) Number of correct performances of the task. (**B**) Performance time in minutes. Error bars represent standard error of means (SEM). These results suggest that, under the stimulation parameters and the cortical target used in the present study, the application of the tDCS protocol did not result in a modulation of performance of a spatial maze task in adults.

The one-way ANOVA conducted to analyze differences between anodal and sham tDCS in the number of correct performances (F_1,29_ = 1, *p* = 0.326, *ηp*^2^ = 0.033) and the total performance time (F_1,29_ = 0.974, *p* = 0.332, *ηp*^2^ = 0.159) revealed no significant differences. Figure 4 depicts the number of correct performances (panel A) and the total performance time (panel B) in both stimulation conditions (anodal vs. sham tDCS).

## 4. Discussion

In our first study we aimed to test the hypothesis of a different influence of visual inputs during spatial processing between children and adults using a MSNT under visual deprivation. In this task we evaluated two parameters of performance efficacy, the number of correct performances in each age group, and total performance time in each single session. To facilitate correct performance without exhausting the attentional and cognitive systems necessary to successfully perform the task, we evaluated performance in two separate sessions. With respect to the number of correct performances, the results showed significantly better performance in children than in adults, and this effect was observed in both sessions. Additionally, no learning effect or significant improvement was observed in session 2, as compared to session 1, in any age group. Thus, although success rates slightly increased and decreased in children and adults, respectively, the number of correct performances was virtually the same between sessions in both groups. Regarding performance times, children were more efficient than adults in both sessions, with differences between participant groups being significant, however, only in session 2. Compared to the first session, performance time was significantly shorter in the second session only in children, which may indicate an improvement effect due to practice in this age group. These findings point to a different impact of visual stimuli on spatial processing according to the specific stage of neural development.

The development of spatial navigation skills is a progressive process influenced by neural maturation, motor development, and their interactions with different sensory experiences, particularly those dependent on sensory modalities such as visual and vestibular [17,52]. Proprioceptive, kinesthetic, and other sensory processing is enhanced in situations of visual deprivation, providing compensatory inputs for spatial navigation [21]. In congenitally blind individuals, it was shown that these compensatory mechanisms have a neuroanatomical foundation, with increased functional connectivity between the occipital lobe and the frontal lobe, the bilateral intraparietal sulcus, the inferior parietal lobe, and the left posterior-to-middle cingulate cortex [20,53]. Thus, complete early visual deprivation leads to adaptive plastic processes of cortical reorganization, which may impact, among others, spatial processing [20,21,53]. This plastic capacity in the early stages of development might also imply that the neuroplasticity resources necessary to compensate for temporary visual deprivation during spatial processing are more versatile and, possibly, effective in childhood than in adulthood [52,54]. This advantage in processing compensatory spatial information and plasticity resources would facilitate the recruitment of alternative or second-level sensory modalities, including auditory, vestibular, proprioceptive, and kinesthetic ones, as well as their interactions, to guide and support navigation and spatial cognition processes under conditions of temporary visual deprivation. In adults, in contrast, this recruitment turns out to be less effective [54,55], although certain cortical mechanisms of egocentric processing during spatial navigation have been shown also in adults [56]. This explanation is consistent with the superior spatial task performance of the children in our study, reflected in a higher number of correct performances and shorter completion times. This means that the greater involvement of complementary multisensory cortical pathways in children may facilitate performance of spatial navigation tasks and other cognitive tasks sensitive to the activation of these compensatory brain mechanisms [18,19,57].

Considering that neuromodulation methods facilitate neuroplasticity processes and associated functions [33], a second aim of our research (study 2) was to explore if increasing cortical excitability of one of the main cortical nodes of egocentric spatial processing, the PPC [32,33,34,35,58], via anodal tDCS, improves spatial navigation in adults under the same visual deprivation applied in study 1. The results indicate that the stimulation protocol did not induce changes in any of the outcome measures, showing a similar number of correct performances and completion times between both stimulation conditions (anodal vs. sham). These findings suggest that, at least under the stimulation parameters applied in our protocol, excitatory anodal tDCS over a principal node of spatial processing does not significatively modify spatial navigation ability in a maze task. A restricted switching between allocentric and egocentric dominance in adults may be the reason why stimulation of an egocentric spatial processing node does not lead to improvements in spatial navigation under visual deprivation. However, considering the high interindividual variability in the neurobiological and functional effects of tDCS in humans [59,60,61,62,63], further tDCS protocols with optimized current intensity, stimulation duration, number of sessions and target area should be explored to analyze the capacity of this neuromodulation technique to improve spatial processing.

Some limitations of study 1 should be noted. Thus, although performance differences between age groups were evident, broadening the age range with multiple stratified age groups might provide further evidence on the critical impact of specific development stages on spatial navigation with visual deprivation and how this is connected with allocentric/egocentric processing. In this connection, since spatial navigation and processing are complex processes, these should be analyzed using multiple tasks and measures to be able to elucidate whether the mechanisms of spatial processing differ depending on the stage of development. Comparing deprivation conditions of different sensory modalities may be useful to understand the contribution of different sensory inputs to spatial processing dependent on the stage of development. Regarding study 2, in addition to the well-known limitation of interindividual variability of non-invasive brain stimulation, limited tDCS focality is another potential shortcoming of the applied stimulation protocol [61,64]. Thus, more focal procedures of stimulation, like HD-tDCS [65], and direct comparisons of the effects of stimulation applied over different cortical targets and nodes of the spatial processing network should be investigated in future studies. Neuroimaging methods concurrent with stimulation may also provide more specific information about the cortical focality of tDCS and might help to identify the specific brain mechanisms associated to the effects of this neuromodulation technique. Based on these methodological adjustments aimed at improving cortical focality and individualizing tDCS doses, stimulation protocols designed to modulate spatial processing might be optimized. Moreover, more effective neuroplasticity changes might be induced implementing multi-session stimulation protocols, which would potentially enhance associated behavioral effects [66]. Likewise, in order to optimize protocols, offline stimulation effects, i.e., tDCS applied before task performance, should be explored given the effects reported in other behavioral paradigms [67], ideally through direct comparisons with the effect of online tDCS. Finally, the limited sample size and variability of brain anatomy between individuals calls for follow up studies to confirm the results of the present study.

## 5. Conclusions

Performance in an MSNT under visual deprivation, as measured with performance time and number of correct performances, is an age-dependent ability, as shown in our study. Children at the age of about 10 years performed a spatial maze task with temporary visual deprivation more efficiently than young adults. Functional access to egocentric spatial navigation strategies in early childhood stages probably reduce the critical relevance of visual inputs for performance of this task in children and promote effective recruitment of other sensory modalities. The activation of the PPC, a principal node of egocentric processing, via excitatory tDCS did not, however, improve spatial navigation in adults under the same conditions of visual deprivation. This might suggest a functional restriction in the access to egocentric processing in adults, who rely on allocentric cues as references for spatial navigation. Optimization of stimulation protocols, tasks and spatial processing measures is necessary to analyze the impact of non-invasive neuromodulation by tDCS on spatial navigation and associated cognitive processes under conditions of sensory deprivation in future. Improving spatial navigation skills and facilitating egocentric processing via neuromodulation might have an impact on the treatment of patients with brain damage and impaired spatial processing, as well as on children with neurodevelopmental disorders.

## Figures and Tables

**Table 1:** Demographic characteristics of the samples.

**Study 1**	
Children group	30
Age	10.97 ± 0.55
Sex	15 girls
Education	5 years
Adults group	30
Age	21.16 ± 1.76
Sex	15 women
Education	13 years
**Study 2**	
Adults	30
Age	21.23 ± 2.01
Sex	15 women
Education	13 years

## Data Availability

The original contributions presented in this study are included in the article. Further inquiries can be directed to the corresponding author.
